# Supplementary material for: Spectrum and Incidence of Adverse Reactions Post Immunization in the Taiwanese Population (2014–2019): An Analysis Using the National Vaccine Injury Compensation Program
Source: Vaccines (Basel). 2024 Oct 3;12(10):1133. doi: 10.3390/vaccines12101133 (PMC11511092; doi:10.3390/vaccines12101133)
Supplement: Supplementary file 1 [file vaccines-12-01133-s001.zip › vaccines-3186238-supplementary.pdf]

Supplementary Materials:

Table S1. Total administered doses of various vaccines included in extended program of immunization in Taiwan from 2010 to 2019.

Vaccine type	Year									
	2010	2011	2012	2013	2014	2015	2016	2017	2018	2019
BCG	150,800	180,679	228,196	194,859	203,652	206,969	121,177	199,342	185,890	178,505
PCV	-	22,638	173,349	647,866	680,537	784,820	660,543	606,407	564,014	540,003
Tdap-IPV/ DTaP-IPV	50,337	135,795	223,778	220,749	215,632	110,844	278,915	200,010	212,356	225,165
DTaP-Hib-IPV	576,146	706,967	829,068	832,374	607,110	798,393	823,205	840,153	766,332	798,393
MMR	395,496	324,100	417,572	447,967	411,203	402,927	416,747	447,218	400,100	411,434
Hepatitis A[b]	8,605	46,963	52,656	63,818	83,635	11,264	12,023	24,840	214,935	395,565
Hepatitis B	440,268	538,475	678,317	603,943	602,178	636,324	627,081	454,066	403,600	529,089
JE [c]	751,141	754,587	767,615	896,924	849,334	828,704	831,265	720,747	608,004	600,255
Varicella vaccine	192,765	165,563	196,913	224,816	194,014	208,433	212,054	204,605	194,959	181,502
PPV23	72,922	105,985	106,930	83,588	85,156	78,100	107,172	125,907	119,049	168,863
Influenza vaccine	2,911,603	2,410,092	2,838,125	2,849,135	2,904,323	2,948,290	6,024,294	5,992,573	5,314,267	5,996,949

[a] From January 2016, the BCG vaccine was scheduled on 5-to-8-month-old instead of after the age of 24 hours.

[b] From January 2018, Hepatitis A vaccine became regular vaccine which was scheduled at the age of 1 year

[c] From May 2018, the regular JE vaccine was changed to live attenuated JE chimeric virus vaccine instead of mouse brain-derived JE vaccine.

The amounts in this table represent the sum of both mouse brain-derived JE vaccine and liver attenuated JE chimeric virus vaccine.

Abbreviations: BCG, Bacillus Calmette–Guérin; PCV, pneumococcal conjugate vaccine; Tdap-IPV, Tetanus toxoid, reduced diphtheria toxoid,

acellular pertussis and inactivated polio vaccine; DTaP-IPV, Diphtheria and tetanus toxoids with acellular pertussis and inactivated polio vaccine; DTaP-Hib-IPV, diphtheria, tetanus, acellular pertussis, *Hemophilus influenzae* type b and inactivated poliovirus vaccine; MMR, measles-mumps-rubella; JE, Japanese encephalitis vaccine; PPV23, 23-valent polysaccharide pneumococcal vaccine

Table S2. Immunization Schedule in Taiwan. (adapted from Taiwan CDC : https://www.cdc.gov.tw/File/Get/83fnbb9oIRBWMBL_AR6Jkw)

Age Vaccine	<24 hr	1 month	2 months	4 months	5 months	6 months	12 months	15 months	18 months	21 months	27 months	5 years
Hepatitis B	HepB1	HepB2				HepB3						
BCG					BCG							
Diphtheria, Tetanus, Pertussis, Hib, Polio			DTaP-Hib-IPV 1	DTaP-Hib-IPV 2		DTaP-Hib-IPV 3			DTaP-Hib-IPV 4			DTaP-IPV

Pneumococcal conjugate vaccine	PCV13 1PCV13 2	PCV13 3				
Varicella		Var				
Measles, Mumps, Rubella		MMR1				MMR2
Japanese Encephalitis[1]			JE1		JE2	
Hepatitis A[2]		HepA1		HepA2		
Influenza				Influenza (yearly)		
Others[3]						

1. The mouse brain-derived vaccine was replaced by the Vero cell-derived Japanese encephalitis vaccine in May 2017.
2. In January 2018, hepatitis A vaccine was introduced into routine immunization program for children born after January 2017. The original hepatitis A vaccine program for children registered in selected indigenous areas has been implemented continuously. In April 2019, hepatitis A vaccine program was expanded to children under 13 years of age from low-income and middle-to-low-income households.
3. Rotavirus vaccine and human papillomavirus vaccine are not included in the routine schedule; they are optional and not government funded. Rotavirus vaccine is introduced since 2-month-old, 2 doses for Rotarix and 3 doses for Rotateq.
